# A prospective longitudinal study of tuberculosis among household contacts of smear-positive tuberculosis cases in Lima, Peru

**DOI:** 10.1186/s12879-016-1616-x

**Published:** 2016-06-08

**Authors:** Larissa Otero, Lena Shah, Kristien Verdonck, Tullia Battaglioli, Timothy Brewer, Eduardo Gotuzzo, Carlos Seas, Patrick Van der Stuyft

**Affiliations:** Instituto de Medicina Tropical Alexander von Humboldt, Universidad Peruana Cayetano Heredia, Av. Honorio Delgado 430, San Martín de Porres, Lima 31 Peru; Department of Public Health, Unit of General Epidemiology and Disease Control, Institute of Tropical Medicine, Antwerp, Belgium; Department of Epidemiology, Biostatistics and Occupational Health, McGill University, Montreal, Canada; Department of Public Health, Unit of Epidemiology and Control of Tropical Diseases, Institute of Tropical Medicine, Antwerp, Belgium; Department of Medicine, David Geffen School of Medicine, University of California, Los Angeles, CA USA; Department of Public Health, Ghent University, Ghent, Belgium

**Keywords:** Multilevel analysis, Household contact investigation, Longitudinal study

## Abstract

**Background:**

Household contacts (HHCs) of TB cases are at increased risk for TB disease compared to the general population but the risk may be modified by individual or household factors. We conducted a study to determine incident TB among HHCs over two years after exposure and to identify individual and household level risk factors.

**Methods:**

Adults newly diagnosed with a first episode of smear-positive pulmonary TB (index cases) between March 2010 and December 2011 in eastern Lima, were interviewed to identify their HHC and household characteristics. TB registers were reviewed for up to two years after the index case diagnosis and house visits were made to ascertain TB cases among HHC. The TB incidence rate ratio among HHCs as a function of risk factors was determined using generalized linear mixed models.

**Results:**

The 1178 index cases reported 5466 HHCs. In 402/1178 (34.1 %) households, at least one HHC had experienced a TB episode ever. The TB incidence among HHCs was 1918 (95%CI 1669–2194) per 100,000 person-years overall, and was 2392 (95%CI 2005–2833) and 1435 (95%CI 1139–1787) per 100,000 person-years in the first and second year, respectively. Incident TB occurred more than six months following the index case’s TB diagnosis in 121/205 (59.0 %) HHCs. In HHCs, bacillary load and time between symptoms and treatment initiation in the index case, as well as the relationship to the index case and the sex of the HHC all had a significant association with TB incidence in HHCs.

**Conclusions:**

Incidence of TB among HHCs was more than ten times higher than in the general population. Certain HHC and households were at higher risk of TB, we recommend studies to compare HHC investigation to households at highest risk versus current practice, in terms of efficiency.

**Electronic supplementary material:**

The online version of this article (doi:10.1186/s12879-016-1616-x) contains supplementary material, which is available to authorized users.

## Background

Household contacts (HHCs) of tuberculosis (TB) patients are at higher risk for TB infection and disease. In recent studies, 4.5 to 7.8 % of HHCs have been found to have active disease [1–3]. Investigating HHCs may facilitate timely diagnosis and treatment of active TB as well as of latent TB infection. However, contact tracing in general and HHC investigation in particular are resource-intensive activities, and their consistent implementation is a challenge in both high- and low-income countries. TB control programs in low-income high-incidence settings face competing priorities [4–6] but limited data suggest that screening HHCs of TB cases for active disease may be cost-effective in low- and middle-income countries [7]. The World Health Organization recently extended its recommendations for HHC tracing to these countries [8].

TB risk in HHCs may vary by individual and household as well as by background community incidence, and investigating all HHCs in high-burden, low-resource countries may be inefficient or unfeasible. Estimates of risk should consider the correlation of risk factors of HHCs within a single household [9] to avoid overestimating effects when ignoring that correlation. To better inform TB programs on the precise burden of TB over time in HHCs, studies with sufficiently long follow-up and with comparable methods need to be conducted [1, 2, 10]. A better understanding of TB risk over time and risk factors would provide evidence to increase efficiency of HHC investigation guidelines in high TB burden settings [6] by strategies and timing following epidemiological data.

Studies have shown a high burden of TB among HHC of multidrug resistant (MDR) TB and drug susceptible cases in Peru [10–13]. We conducted a prospective study to determine the TB incidence over 2 years among HHC of all new TB cases in eastern Lima, and to identify individual and household level risk factors that determine this incidence.

## Methods

### Design, setting and study population

We conducted a longitudinal prospective study of TB incidence among HHCs of smear-positive cases in an eastern Lima district. The district is the most inhabited in Peru with 1,047,026 inhabitants in 2013 of which 27 % are living in poverty, higher than the 17.5 % for Lima [14]. TB incidence in the district in 2012 was 175 cases of TB per 100,000 inhabitants, and among those patients tested for HIV 2.9 % were positive. The district has 34 public health facilities where clinics implementing the directly observed treatment short-course strategy (DOTS) operate. Consenting adults diagnosed with a first episode of smear-positive pulmonary TB between March 2010 and December 2011 (index cases) were enrolled in the present study.

Peruvian guidelines recommend evaluation of HHCs (symptoms screening, tuberculin skin test and chest X-ray) at diagnosis of the index case, and at the second and sixth month of the index case treatment. Isoniazid prophylaxis for TB is recommended to all HHCs under five years old and to HHC from 5 to 19 years old with latent tuberculosis infection documented with a positive tuberculin skin test. The present study did not interfere with routine HHC investigation by National TB Program health center staff. In 2010–2011, the study district reported that 74 % of HHCs had been evaluated and that prophylaxis had been prescribed to 54 % of eligible HHCs.

### Baseline data collection and longitudinal detection of TB among HHCs

At enrollment, trained research field workers interviewed index cases to collect demographic and HHC data using a structured questionnaire. Index cases provided the names of their HHCs and any previous TB episodes among them. To determine incident TB for known HHCs, district TB registers were reviewed periodically for up to two years after the diagnosis of the index case. In each of these reviews, the names of newly registered TB cases were compared to the names of the HHC reported by index cases to capture TB diagnoses among HHCs. At the end of the study, the database of HHC reported by the index cases was matched with the TB registers in order to capture HHC registered as a TB case but missed by the periodic visual inspection. Queries were run to match combinations of given and family names and initials, thus reducing the risk of missing matches. To confirm true matches, two authors (LO and KV) subsequently verified all hits.

To capture TB diagnoses outside the study district or in a private health facility, a single household visit was conducted at the end of the two-year follow-up period. During that visit, the index case or the head of the household was asked if any of the HHC had developed TB since the index case diagnosis. Households where the index case had defaulted treatment or died did not receive the final follow-up visits. This was the case for 12.8 % (151/1178) of the study households.

### Study definitions

A HHC was defined as a person sleeping under the same roof and sharing cooking facilities with the index case for at least three months before the case’s diagnosis. Past TB in an HHC was defined as a TB episode occurring more than two years before the diagnosis of the index case; recent TB was defined as TB diagnosed within the two years before the diagnosis in the index case. Incident TB in an HHC was defined as TB that came to diagnosis up to two years following the diagnosis of the index case.

### Analysis

Data were entered in an Access (Microsoft Redmond, WA, USA) database and analyzed with Stata v.12 (Stata Corp, 12.0, College Station, TX).

Index cases who lived in congregate settings such as shelters or rehabilitation centers for addicts and those who lived alone were excluded from the analysis. The contact’s, household and index case’s characteristics of HHC with incident TB were compared to those of HHC without an episode of TB. As our data source for incident TB episodes among HHC was the district TB register, which was checked up to two years after the inclusion of the last index case, complemented with a final household visit, we considered the two years follow up complete for all HHC. Only those that developed incident TB were censored.

Risk factors for acquiring TB, including those not measured directly, may be correlated at household/index case level. To account for this correlation, we used generalized linear mixed models (GLMM) with random effects for household, in Poisson regression analysis for count outcomes. A simple logistic regression model may overestimate the effects measured. The outcome was the number of HHCs with incident TB. To protect against the impact of model-misspecification, robust standard errors were used. Covariates that were significant (at *p* < 0.2) in the bivariate analysis as well as those that were a priori expected to have an influence on or to be confounding the incidence of TB in HHC were considered for inclusion in the model. Models were compared by backward selection. Variables with the weakest association were taken out until a significant difference between two models was found.

## Results

### Study population

The study enrolled 1295 participants with a first diagnosis of smear-positive TB. Of these, 117 (9.0 %) were excluded: 30 were HHCs of a previously enrolled case, 59 lived alone and 28 lived in congregate settings. Included and excluded index cases were similar in age, but a higher proportion of excluded participants were male (81.0 % vs. 59.2 %) (*p* < 0.001). The 1178 (91 %) index cases included in the analysis reported 5466 HHCs (median number per index case = 4, interquartile range (IQR) 3–6). Table 1 shows the characteristics of the index cases, of the household and of the HHCs.

**Table 1:** Characteristics of the index cases and household contacts

Characteristic	N (%)^a^
Index case	
Male sex	481 (40.0)
Age (median, IQR) in years	26 (21–35)
Presence of cough	1135 (96.4)
Time from symptoms to treatment initiation (in days)	33 (19–62)
Smear positivity	
Scanty	40 (3.4)
+/++	562 (47.7)
+++	576 (48.9)
Treatment	
For drug susceptible TB	1162 (98.6)
For MDR TB	16 (1.4)
Tobacco use	
None	713 (60.7)
	416 (35.4)
	46 (3.9)
HIV	
Positive	884 (75.0)
Negative	18 (1.5)
Not tested	276 (23.4)
Household characteristics	
Past TB episodes	62 (5.3)
Recent TB episodes	29 (2.5)
Persons per room	2.1 (1.5–3.0)
Household contact characteristics (*n* = 5466)	
Male sex	2674 (48.9)
Age (median, IQR) in years	24 (12–40)
Relationship with the index case	
Sibling	1416 (25.9)
Parent	946 (17.3)
Partner	437 (7.9)
Offspring	1031 (19.9)
Other	1636 (29.9)

^a^ frequencies are presented in absolute number and percentages, except for continuous variables where the median and the interquartile range (IQR) is presented. *TB* tuberculosis, *MDR TB* multi drug resistant TB

For smear positivity: scanty = 1–9 acid fast bacilli (AFB) in 100 fields, +/++ = from 10 AFB in 100 fields to 10 AFB per field in at least 50 fields, +++ = more than 10 acid fast bacilli in at least 20 fields. A TB episode in a HHC more than two years before the diagnosis of the index case, d = a TB episode within two years before the index case diagnosis

### TB burden in households and among HHCs

**Fig. 1:**
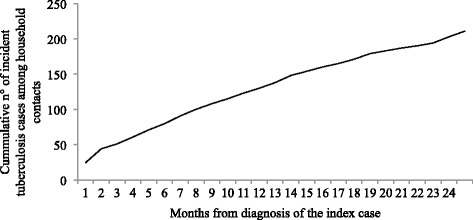
Incident tuberculosis cases among household contacts by month

Of the HHCs, 602 (11.0 %) HHC had experienced at least one TB episode –either past, recent or incident. Thirty (0.6 %) HHC had more than one episode. Past TB was reported for 292 (5.3 %) HHCs, recent TB for 135 (2.5 %) HHC, 205 (3.8 %) had incident TB. The median time between the diagnosis of a recent TB episode in a HHC and the diagnosis of the index case was 275 days (IQR, 86–483). Table 2 shows the burden of TB among HHCs per household: in 402/1178 (34.1 %) households, at least one HHC had a TB episode at some point and, in 163 (13.8 %) households, at least one incident TB episode occurred during the follow up period. Incident TB episodes occurred among 3.8 % of HHC, which corresponds to 1918 (95%CI 1669–2194) incident TB episodes per 100,000 person-years. The TB incidence rate among HHCs was 2392 (95%CI 2005–2833) per 100,000 person-years in the first year and 1435 (95%CI 1139–1787) per 100,000 person-years in the second year. The median time between the index case diagnosis and incident TB episode was 256 days (8.4 months, IQR, 94–455). Of the 673 HHCs ≤ 5 years old, 16 (2.4 %) developed incident TB. Figure 1 shows the cumulative number of incident TB; 121/205 diagnoses (59.0 %) occurred beyond the sixth months following the index cases’ enrollment.

**Table 2:** Past, recent and incident tuberculosis episodes among household contacts, per household

Number of tuberculosis episodes in household contacts	N° households	% households
≥1 past case only	149	12.6
≥1 recent case only	64	5.4
≥1 incident case only	105	8.9
≥1 past + ≥1 recent case	26	2.2
≥1 past + ≥1 incident case	35	3.0
≥1 recent + ≥1 incident case	17	1.4
≥1 past + ≥1 recent + ≥ 1 incident case	6	0.5
No cases	776	65.9
Total	1178	100

Past case = household contact that had a tuberculosis episode more than two years before the diagnosis of the index case. Recent case = household contact that had a tuberculosis episode in the two years previous to the diagnosis of the index case. Incident TB case = TB episode in a household contact after the diagnosis of the index case

### Household contacts characteristics associated to incident TB in HHC

At individual contact level, relationship to the index case, sex and age were associated with incident TB in HHCs in bivariate analysis (Table 3). To determine if young children of mothers with TB had a higher risk of TB, we looked for an interaction of the age of the HHC and the index case being a mother or not, but found none.

**Table 3:** Incident tuberculosis in 5466 household contacts (HHC) in function of the HHC’s characteristics

	Incident TB in the HHC		Crude odds ratio (95%CI)
	Yes	No	
Age in years			
≤5	16 (2.4 %)	657 (97.6 %)	1.0 (0.5–1.8)
6–19	56 (3.8 %)	1407 (96.2 %)	1.5 (1.0–2.5)
20–40	100 (5.0 %)	1883 (95.0 %)	2.1 (1.4–3.2)
≥41	33 (2.5 %)	1314 (97.6 %)	1
Sex			
Female	88 (3.2 %)	2704 (96.9 %)	1
Male	117 (4.4 %)	2557 (95.6 %)	1.4 (1.1–1.9)
Relationship			
Sibling	84 (5.9 %)	1332 (94.1 %)	2.9 (2.0–4.4)
Parent	21 (2.2 %)	925 (97.8 %)	1.0 (0.6–1.8)
Partner	27 (6.2 %)	410 (93.8 %)	3.0 (1.8–5.0)
Offspring	38 (3.7 %)	993 (96.3 %)	1.8 (1.1–2.8)
Other	35 (2.1 %)	1601 (97.9 %)	1
Past TB			
Yes	14 (4.8 %)	278 (95.2 %)	1.3 (0.7–2.3)
No	191 (3.7 %)	4983 (96.3 %)	1
Recent TB			
Yes	6 (4.4 %)	129 (95.6 %)	1.2 (0.5–2.7)
No	199 (3.7 %)	5132 (96.3 %)	1

*TB* tuberculosis, *HHC* household contact, *CI* confidence interval

### Index case and household characteristics associated to incident TB in HHC

Bivariate analysis for household and index case risk factors is shown in Table 4 (1178 households). Time from symptoms to treatment in the index case, sputum bacillary load and a past TB episode in any member of the household were associated with incident TB in HHCs. Tobacco use, education, working status and marital status of the index case and socio-economic level of the household were not associated with incident TB (data not shown).

**Table 4:** Incident tuberculosis among household contacts in 1178 households in function of the index case and household characteristics

	Incident TB in the household		Crude odds ratio (95%CI)
	Yes	No	
Index case characteristics			
Sex			
Female	86 (12.3 %)	611 (87.7 %)	0.7 (0.5–1.0)
Male	77 (16.0 %)	404 (84.0 %)	1
Age			
18–27	96 (14.4 %)	571 (85.6 %)	1.0 (0.6–1.5)
28–37	34 (14.2 %)	206 (85.8 %)	0.7 (0.4–1.4)
38–47	11 (11.0 %)	89 (89.0 %)	0.9 (0.5–1.4)
>48	22 (13.8 %)	149 (87.1 %)	1
Cough			
Yes	160 (14.1 %)	975 (85.9 %)	1
No	3 (7.0 %)	40 (93.0 %)	0.5 (0.1–1.5)
Time from symptoms to treatment^a^			
0–30 days	50 (10.0 %)	448 (90.0 %)	1
>30 days	113 (16.6 %)	567 (83.4 %)	1.8 (1.3–2.5)
Smear positivity at diagnosis^b^			
Scanty	1 (2.5 %)	39 (97.5 %)	0.2 (0.0–1.3)
+/++	110 (12.7 %)	755 (87.3 %)	1
+++	52 (19.1 %)	221 (80.9 %)	1.6 (1.1–2.3)
Treatment type			
Drug sensitive	159 (13.7 %)	1003 (86.3 %)	0.5 (0.2–1.5)
Multidrug resistant	4 (25.0 %)	12 (75.0 %)	1
Household characteristics			
Past TB episodes^c^ in the household			
Yes	41 (19.0 %)	175 (81.0 %)	1.6 (1.1–2.4)
No	122 (12.7 %)	840 (87.3 %)	1
Recent TB episodes^d^ in the household			
Yes	23 (20.4 %)	90 (79.7 %)	1.7 (1.0–2.8)
No	140 (13.2 %)	925 (86.9 %)	1
Persons per room (median, IQR)	2.5 (1.8–3.8)	2 (1.5–3)	1.1 (1.0–1.2)

^a^ = time from symptoms was calculated from the date the participant reported to start coughing until the date of treatment registered, ^b^scanty = 1–9 acid fast bacilli (AFB) in 100 fields, +/++ = from 10 AFB in 100 fields to 10 AFB per field in at least 50 fields, +++ = more than 10 acid fast bacilli in at least 20 fields,smear positivity was classified following standard national TB program guidelines^13^, ^c^ = a TB episode in a HHC more than two years before the diagnosis of the index case, ^d^ = a TB episode within two years before the index case diagnosis

### Multilevel analysis of index case, household and household contacts characteristics associated to incident TB in HHC

Table 5 shows the results of the multivariate analysis and the Additional file 1 shows the bivariate analysis of additional characteristics. Bacillary load and the time from symptoms to treatment of the index case, as well as the relationship with the index case and the sex of the HHC remained significantly associated with incident TB in HHCs. No household characteristics studied were significantly associated with incident TB. The standard deviation of the between-household difference on the effect of the incidence rate ratio of TB was 1.4, 95%CI 1.1-1.9. This finding suggests that, in addition to the characteristics studied, unmeasured covariates specific to each index case/household also affect TB incidence in HHCs.

**Table 5:** Index case, household and household contacts characteristics and their relation to incident tuberculosis among household contacts

	Bivariate GLMM analysis		Multivariate GLMM analysis	
	IRR	95%CI	IRR	95%CI
Index case characteristics				
Sex				
Female	1			
Male	0.7	0.5–1.0	-	
Age				
18–27	1.3	0.8–2.3	-	
28–37	1.6	0.8–2.9	-	
38–47	1.1	0.5–2.6	-	
>48	1			
Cough				
Yes	1			
No	0.5	0.1–1.8	-	
Time from symptoms to treatment^a^				
0–30 days	1		1	
>30 days	1.9	1.3–2.8	1.8	1.2–2.7
Smear positivity at diagnosis^b^				
scanty	0.2	0.02–1.4	0.2	0.03–1.7
+/++	1		1	
+++	1.6	1.1–2.4	1.5	1.02–2.3
Treatment type				
Drug sensitive	1			
Multidrug resistant	1.6	0.5–5.4	-	
Household characteristics				
Past TB episodes^c^ in the household				
Yes	1.2	0.6–2.1	-	
No	1			
Recent TB episodes^d^ in the household				
Yes	1.2	0.5–2.8	-	
No				
Persons per room	1.01	0.9–1.1	-	
Household contact characteristics				
Sex				
Female	1		1	
Male	1.5	1.1–1.9	1.4	1.1–1.9
Age				
0–5	1.0	0.5–1.8	0.9	0.4–1.9
6–19	1.7	1.1–2.6	1.2	0.7–2.3
20–40	2.3	1.5–3.5	1.5	0.9–2.6
>40	1		1	
Relationship				
Sibling	2.8	1.8–4.3	2.6	1.6–4.0
Parent	0.9	0.5–1.6	1.1	0.5–2.3
Partner	2.9	1.7–5.0	2.7	1.5–4.8
Offspring	1.5	0.9–2.5	1.5	0.9–2.4
Other	1		1	

*GLMM* generalized linear mixed models, *IRR* incidence rate ratio, *CI* confidence interval

^a^ = time from symptoms was calculated from the date the participant reported to start coughing until the date of treatment registered, ^b^scanty = 1–9 acid fast bacilli (AFB) in 100 fields, +/++ = from 10 AFB in 100 fields to 10 AFB per field in at least 50 fields, +++ = more than 10 acid fast bacilli in at least 20 fields, ^c^ = a TB episode in a HHC more than two years before the diagnosis of the index case, ^d^ = a TB episode within the two year

Among the 287/5466 HHCs who had three of the above determinants –excluding male sex- associated to incident TB (siblings/partners, index case with a time from symptoms to treatment of > 30 days and a high bacillary load), 29 (10.1 %) had incident TB; among the 2419 HHC that had two of those factors, 159 (6.6 %) had incident TB.

## Discussion

The incidence of TB in HHC of new smear-positive TB cases in this district was 1918 per 100,000 person-years, ten times higher than that for the general population in the district. Among incident TB cases, 59 % occurred beyond six months of the index case’s diagnosis and would not have been identified as by current HHC investigation contracting procedures. TB burden in households was high, with 34 % of households having a HHC with past, recent or incident TB in addition to the index case. Being a sibling or a partner of the index case, and more than 30 days between symptoms treatment initiation or a high bacillary load in the index cases were associated with incident TB.

Index cases included in the study were representative of the new TB cases in the district, but this study has some limitations. Incident TB in HHC could have been diagnosed outside the district or in facilities not managed by the Ministry of Health and not appear in the TB register. We conducted household visits to ascertain these cases. However, households of index cases who defaulted treatment or died were not visited. This could lead to underestimating TB incidence in HHC. Furthermore, our definition of incidence was based on diagnosis and case notification. Since only individuals with symptoms are likely to present for diagnosis, true incidence may be underestimated. Past and recent TB episodes might also be underestimated as these were reported by the index case. Strengths of this study include its prospective design, the use of two complementary follow up approaches to ascertain incident TB in HHC and accounting for the correlation of data at household level in the analysis.

In a systematic review of 25 studies in low- and middle-income countries, the weighted estimated average of incident TB in HHC in the first year of follow-up was 478 (95%CI 897–2427) per 100,000 person-years and 831 (95%CI 624–1106) per 100,000 person-years in the second year [1]. We found rates approximately two and five times higher, respectively. Some studies conducted in Lima have determined incident TB among HHCs of selected TB cases. In the first and in the second year, 2360 incident TB episodes per 100,000 person-years occurred among HHC of 80 MDR-TB index cases in southern Lima [10], while a more recent study found 2456 incident TB episodes per 100,000 person-years occurred among HHC of 213 multidrug resistant TB cases and 4351 incident TB episodes per 100,000 person-years among HHC of 487 drug susceptible index cases [13]. A retrospective study that included 693 MDR and extensively drug resistant TB index cases found 3165 and 1092 incident TB cases in HHC per 100,000 person-years in the first and second year, respectively [12]. Our data demonstrate similar high rates of incident TB among HHCs of a large sample of unselected new pulmonary TB patients.

Our findings confirm that siblings and partners [15, 16], male HHC [10, 16], and HHC exposed to index cases with higher sputum bacillary loads [13] or that are infectious for a longer period [17] are more likely to develop incident TB. Contrary to what is expected, HHC under six years old were not found to be at higher risk for incident TB than older age groups. However, it is possible that TB in children is under diagnosed in Peru. One explanation consistent with our findings is that there are important effects at the household/index case level on the incidence of TB in HHCs. This was also found in a multilevel study in Pakistan that looked into household and HHC determinants of TB infection [9]. Characteristics at household level that may have influence should be considered in future studies, such as ventilation, sleeping arrangements, frequent exposure to non-household members as well as genetic predisposition of families. Measuring these potential risk factors might strengthen of the index case/household association on incidence rate ratio of TB among HHCs. Prioritizing individuals at highest risk for HHC investigation could reduce the number of HHC to be screened, but such selection may be logistically cumbersome. In addition, staff, patients and HHC themselves may not find it acceptable. However, it could be an option to use index case characteristics that put households at higher risk in order to select complete households in which to screen all contacts.

While HHCs of active TB patients may have a much higher risk of TB than the general population, TB cases in HHCs still account for only a small proportion of the total TB burden in a medium and high incidence area [18]. For example, the 205 incident cases found in our study corresponded to approximately 5 % of the district’s TB notifications during the same period. Furthermore, in some high incidence areas, community transmission can be higher than household transmission [19, 20]. The background TB incidence at which full HHC contact investigation becomes cost-effective as compared to investigating only selected high-risk HHC or index cases/households would provide valuable information for National TB Programs decision-making.

Finally, National TB Programs typically attempt to evaluate HHC at the start of the index case’s TB treatment, at the change of their treatment phase and at the end of their treatment. However, this approach does not take into consideration the time that may elapse occur between TB infection and disease [1, 18, 20]. For that reason, even full compliance to Peruvian guidelines for HHC investigation of drug sensitive cases would have failed to identify 59 % of incident TB episodes as HHCs.

## Conclusions

This study demonstrated that HHC of TB cases in Lima, Peru are at very high risk for incident TB. Studies comparing the feasibility and cost effectiveness of all-inclusive versus risk based HHC investigation strategies within National TB Programs are needed to inform policy formulation.

## Abbreviations

CI, confidence interval; GLMM, generalized linear mixed models; HHC, household contact; IRR, incidence rate ratio; TB, tuberculosis

## Additional file

Additional file 1: Additional index case characteristics and their relation to incident tuberculosis among household contacts. (DOCX 18 kb)
