# Supplementary material for: A prospective longitudinal study of tuberculosis among household contacts of smear-positive tuberculosis cases in Lima, Peru
Source: BMC Infect Dis. 2016 Jun 8;16:259. doi: 10.1186/s12879-016-1616-x (PMC4898451; doi:10.1186/s12879-016-1616-x)
Supplement: Additional file 1: — Additional index case characteristics and their relation to incident tuberculosis among household contacts. (DOCX 18 kb) [file 12879_2016_1616_MOESM1_ESM.docx]

Additional file 1: Additional index case characteristics and their relation to incident tuberculosis among household contacts.

|  | Bivariate GLMM analysis | |
| --- | --- | --- |
|  | IRR | 95%CI |
| Working status |  |  |
| Student | 1 |  |
| Working | 1.5 | 0.7-3.4 |
| Unemployed | 1.9 | 0.9-3.9 |
| Marital status |  |  |
| Single | 1 |  |
| Married/cohabiting | 0.8 | 0.5-1.6 |
| Divorced/widowed | 1.1 | 0.6-2.0 |
| Tobacco use |  |  |
| Never | 1 |  |
| Used to smoke / smokes | 0.6 | 0.3-1.3 |
| Diabetes mellitus |  |  |
| No | 1 |  |
| Yes | 0.7 | 0.3-1.6 |
| Education level^a^ |  |  |
| Primary | 1 |  |
| High school | 0.9 | 0.6-1.2 |
| Higher education | 0.9 | 0.6-1.4 |
|  |  |  |
| HIV |  |  |
| Negative | 1 |  |
| Positive | 0.5 | 0.1-2.2 |
| Not done | 0.7 | 0.4-1.2 |
| Body mass index at diagnosis | 1.0 | 0.9-1.1 |
| Socio economic status |  |  |
| Not poor | 1 |  |
| Poor | 1.5 | 0.9-2.2 |

GLMM: generalized linear mixed models, IRR: incidence rate ratio, CI: confidence interval. **^a^** = highest level achieved
